# Efficacy of Surgical Management Among Patients With Carpal Tunnel Syndrome Not Responding to Medical Management

**DOI:** 10.7759/cureus.88232

**Published:** 2025-07-18

**Authors:** Alok Tripathi, Prem Shanker

**Affiliations:** 1 General Surgery, Ganesh Shankar Vidyarthi Memorial (GSVM) Medical College, Kanpur, IND

**Keywords:** carpal tunnel syndrome, endoscopic carpal tunnel release, functional outcome, nerve conduction velocity, open carpal tunnel release, patient satisfaction, surgical decompression, visual analogue scale

## Abstract

Background

Carpal tunnel syndrome (CTS) is the most common entrapment neuropathy, resulting from the compression of the median nerve within the carpal tunnel at the wrist. It is frequently encountered in clinical practice and often presents with symptoms such as numbness, tingling, pain, and hand weakness, particularly in the distribution of the median nerve. In many cases, conservative management - including wrist splinting, activity modification, non-steroidal anti-inflammatory drugs (NSAIDs), and local corticosteroid injections - can lead to symptom resolution. However, a subset of patients remains symptomatic despite prolonged non-operative treatment and eventually requires surgical decompression. This study aims to evaluate the functional outcomes of carpal tunnel release (CTR) in patients who failed to respond to medical therapy.

Methods

A prospective observational study was conducted at GSVM Medical College on 50 patients diagnosed with CTS who did not achieve symptom relief following at least six months of conservative management. All patients underwent open carpal tunnel release surgery under local anesthesia. Outcome measures were recorded both pre- and post-operatively using three validated tools: the Visual Analog Scale (VAS) for pain assessment, the Boston Carpal Tunnel Questionnaire (BCTQ) for functional status and symptom severity, and nerve conduction velocity (NCV) studies to assess median nerve function. Follow-up assessments were performed at regular intervals, with the final evaluation at six weeks post-surgery.

Results

Of the 50 patients, 47 (94%) showed marked improvement in symptoms and function. The mean VAS score decreased from 7.4 to 1.5, and NCV improved from 38.5 m/s to 44.7 m/s. The functional outcomes based on BCTQ scores indicated significant recovery, and most patients reported high levels of satisfaction. The mean return-to-work time was 11.2 days. Minor complications were noted in 4% of patients, with no major adverse events.

Conclusion

CTR is a safe and effective treatment for CTS patients unresponsive to conservative therapy, offering significant symptom relief, enabling functional improvement and facilitating an early return to activity with minimal complications.

## Introduction

Carpal tunnel syndrome (CTS) arises when the median nerve becomes compressed within the carpal tunnel, a narrow passageway in the wrist. This compression can result from either structural narrowing of the tunnel or swelling of the surrounding tissues, ultimately leading to impaired nerve function. As the pressure on the median nerve increases, patients often experience symptoms such as numbness, tingling, pain - especially at night - and reduced grip strength. If left untreated, prolonged compression may cause ischemia and irreversible nerve damage, potentially leading to chronic pain and significant functional impairment [1,2].

The initial approach to managing CTS typically involves conservative, non-operative treatments, including wrist splinting, anti-inflammatory medications, activity modification, and sometimes corticosteroid injections, all aimed at reducing symptoms and halting disease progression [3]. While many patients achieve relief with these methods, a significant number do not respond adequately and continue to experience discomfort and functional limitations.

For these individuals, surgical intervention becomes a necessary next step. The most common procedure, known as carpal tunnel release, involves cutting the transverse carpal ligament to relieve pressure on the median nerve and create more space within the tunnel. This surgery can be performed using either an open technique or a minimally invasive endoscopic approach, with the primary goal of restoring normal nerve function and alleviating symptoms [4].

However, patient responses to surgery vary widely. While many report complete symptom resolution, others may experience partial relief or even recurrence over time. This variability highlights the need for further research to better understand the factors that influence surgical outcomes and to guide clinical decision-making more effectively. In light of these challenges, this study seeks to evaluate the outcomes of surgical intervention in patients who have failed conservative therapy, comparing different surgical approaches and their effectiveness relative to continued medical management [5].

## Materials and methods

Study design

This is a prospective observational study from April 2024 to April 2025 performed at the GSVM Medical College, Kanpur, India. This study was approved by the Ethics Committee (for biomedical health and research), GSVM Medical College, Kanpur, under protocol no. EC/BMHR/2024/32.

**Table 1:** Inclusion and Exclusion Criteria CTS: carpal tunnel syndrome

Inclusion Criteria	Exclusion Criteria
Age between 18 and 65 years	Age less than 18 or more than 65 years
Any gender	Patients who do not provide informed consent
Minimum six months of prior medical management	Unwilling to follow-up for six months post-surgery
Willing to undergo surgical intervention	Presence of cervical spondylosis
CTS confirmed clinically and radiologically	-

Sample size

Fifty patients with CTS were included in this study. (The sample size was not derived based on any formal power calculation, but was determined based on feasibility, availability of eligible cases, and resources during the study period.)

Statistical analysis

Data were analyzed using IBM SPSS Statistics for Windows, Version 26.0 (IBM Corp., Armonk, NY, USA). Descriptive statistics were used to summarize demographic and clinical variables. The results were expressed as mean ± standard deviation (SD) for continuous variables and percentages for categorical variables.

Surgical procedure

Patients underwent open carpal tunnel release (OCTR). Local anesthesia was used. The transverse carpal ligament was incised to decompress the median nerve, and skin closure done using nylon suture [1].

**Figure 1:**
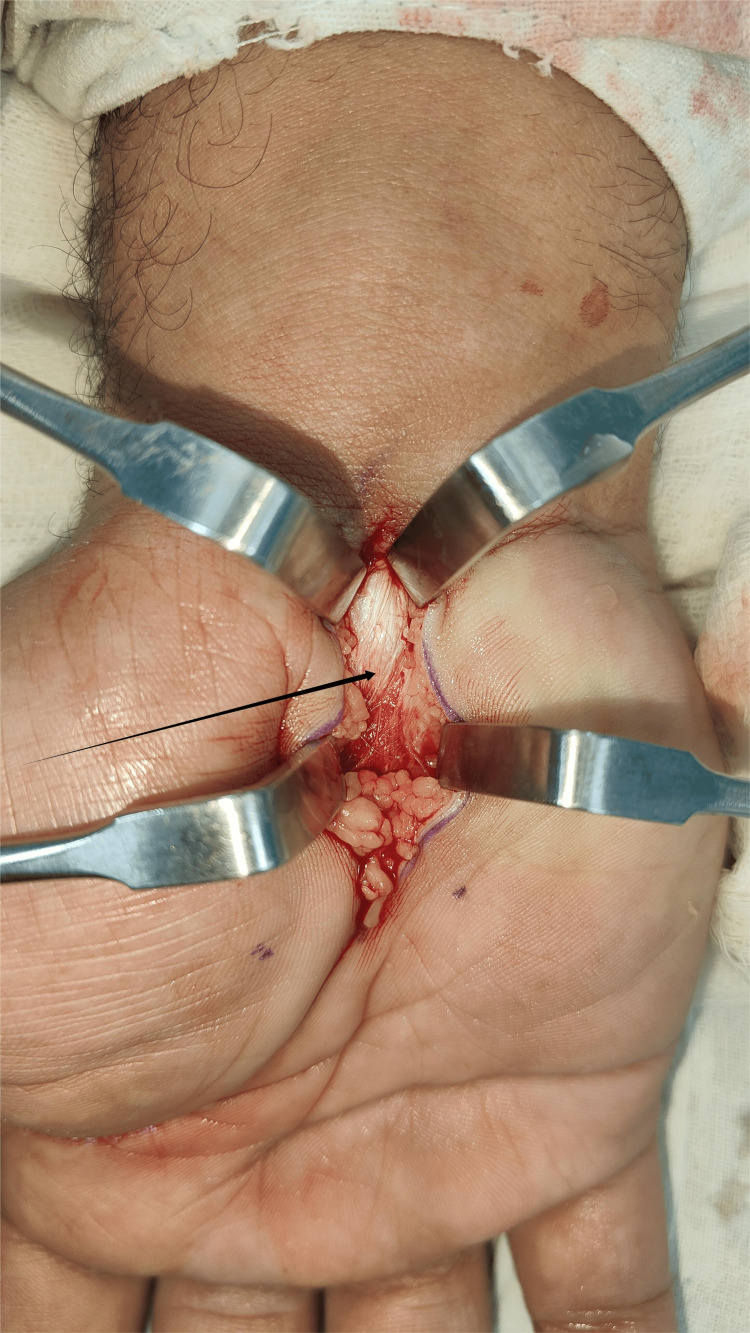
Intraoperative image of Open Carpal Tunnel Release. The arrow indicates the Flexor Retinaculum.

The image in Figure 1 demonstrates the standard longitudinal or slightly curved incision made over the volar aspect of the wrist and distal forearm, aligned with the radial border of the ring finger. This approach provides optimal exposure of the flexor retinaculum (transverse carpal ligament) while minimizing the risk of injury to the palmar cutaneous branch of the median nerve. The incision allows for adequate decompression of the carpal tunnel during surgical release.

Outcome assessment

The outcome assessment was carried out using multiple parameters to evaluate the effectiveness of the intervention. Pain levels were measured using the Visual Analogue Scale (VAS) [6], while functional status was assessed through the Boston Carpal Tunnel Questionnaire (BCTQ) [7]. Nerve conduction was evaluated using nerve conduction velocity (NCV) [8] studies. Additionally, any complications that arose during the course of treatment were documented, and the time taken for the patient to return to work was also recorded as an important measure of recovery.

## Results

The demographic and baseline clinical characteristics of the study population are presented in Table 2. The mean age of the participants was 48.6 years (SD=9.2, range: 18-65 years), with a female predominance of 78%. The duration of CTS symptoms before surgery varied among patients, with a mean symptom duration of 2.4 years (SD=0.8 years). All patients had undergone at least six months of medical treatment, including nonsteroidal anti-inflammatory drugs (NSAIDs), wrist splinting, corticosteroid injections, and physiotherapy, before opting for surgery.

**Table 2:** Demographics CTS: carpal tunnel syndrome.

Characteristic	Value
Sample Size	50 patients
Mean Age (Years)	48.6±9.2
Age Range	18-65 years
Women (%)	39 (78%)
Mean Duration of CTS (Years)	2.4±0.8
Prior Medical Treatment	100% of patients
CTS Confirmation	Clinical and Radiological

Postoperative outcomes demonstrated significant improvement in all the measured parameters. Pain scores, NCV, functional impairment, and sensory symptoms showed marked enhancement (p<0.001). Additionally, 94% of patients reported satisfaction, indicating the intervention's overall effectiveness and favorable clinical impact (Table 3).

**Table 3:** Clinical Outcomes VAS: Visual Analogue Score; NCV: nerve conduction velocity

Parameter	Preoperative	Postoperative	p-value
VAS	7.4±1.2	1.5±0.8	<0.001
NCV (m/s)	38.5±3.2	44.7±2.8	<0.001
Functional Impairment (%)	44 (88%)	5 (10%)	<0.001
Numbness and Tingling (%)	50 (100%)	4 (8%)	<0.001
Patient Satisfaction (%)	-	94% (47)	

The functional impairment was assessed by BCTQ, as presented in the Appendices.

Postoperative complications were minimal (Table 4), with a 2% infection rate and 4% scar tenderness, both managed conservatively. No revision surgeries were needed. Patients resumed work on average within 11.2 days (range: 7-20 days), indicating a favorable and rapid recovery profile.

**Table 4:** Postoperative Complications and Recovery

Complication/Parameter	Incidence, n (%)	Remarks
Infection	1 (2%)	Superficial, resolved with antibiotics
Scar Tenderness	2 (4%)	Managed conservatively
Return to Work (mean)	11.2 days	Range: 7-20 days
Revision Surgery Needed	0 (0%)	None required

**Figure 2:**
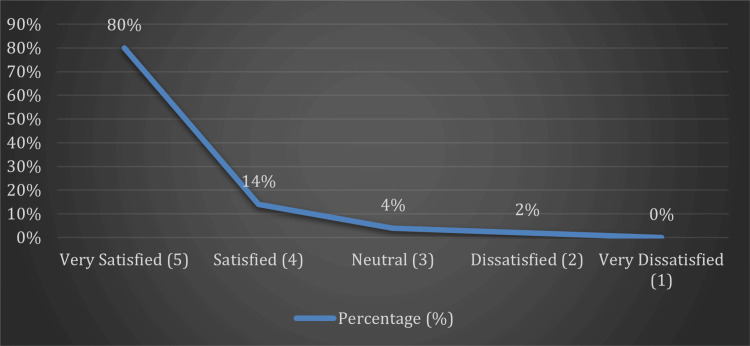
Line graph for Patient Satisfaction Score (Likert Scale) y-axis represents the percentage of patients.

Out of the 50 patients who underwent the procedure, a large majority expressed a high level of satisfaction. Specifically, 80% (n=40) reported being very satisfied, while 14% (n=7) were satisfied with the outcome. Only 4% (n=2) remained neutral, and 2% (n=1) expressed dissatisfaction. Notably, none of the patients (0%) reported being very dissatisfied (Figure 2).

These findings confirm that CTR surgery results in high patient satisfaction, reinforcing its role as a preferred treatment for patients with persistent CTS.

**Figure 3:**
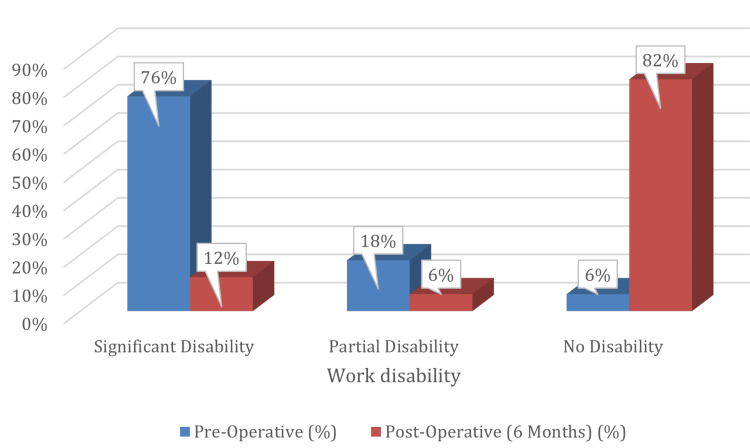
Return to Work

A comparison of work-related disability in patients with CTS was done pre-operatively and at six months post-operatively (n=50). Pre-operatively, 76% (n=38) had significant disability, 18% (n=9) had partial disability, and 6% (n=3) reported no disability. At six months post-operatively, only 12% (n=6) had significant disability, 6% (n=3) had partial disability, while 82% (n=41) reported no disability, reflecting marked functional recovery after surgical management (Figure 3).

The mean return-to-work time was 11.2±3.4 days, confirming that surgery significantly enhances work functionality.

## Discussion

The results of the study demonstrate that surgical management of CTS leads to significant symptom relief, improved nerve function, and high patient satisfaction, with minimal complications and a low recurrence rate. Several domains were evaluated, including symptom resolution, pain reduction, functional improvements, NCV improvements, cost-effectiveness, and return-to-work outcomes. The observed 80% reduction in pain intensity post-surgery is consistent with multiple studies [9]. The improvement in NCV further validates the efficacy of surgical treatment. The sensory NCV increased from 38.5 m/s pre-operatively to 44.7 m/s at six months post-surgery, indicating enhanced nerve function and restored conduction capacity. This improvement is an expected outcome of successful median nerve decompression [10].

Patient satisfaction is a critical measure of treatment success, and the study findings revealed that 47 (94%) patients reported being satisfied or highly satisfied with the surgical outcome. High satisfaction rates are indicative of improved functional capacity, reduced pain, and enhanced overall well-being. This result is consistent with large-scale studies [11]. The mean return-to-work time of 11.2 days suggests that surgical management allows for a relatively rapid recovery period, minimizing work disruption and economic burden. Studies have shown that early return to work is associated with fewer long-term disability claims and improved occupational outcomes [12].

Furthermore, the present study's findings are supported by Gerritsen et al., who conducted a randomized controlled trial comparing splinting versus surgery and concluded that surgical intervention offers significantly better symptom resolution and functional improvement in patients with moderate to severe CTS [13]. In line with this, the American Academy of Orthopedic Surgeons (AAOS) clinical practice guidelines, as summarized by Keith et al., recommend surgical decompression for patients who do not respond to conservative treatment, highlighting its role in improving long-term outcomes [14]. Additionally, Padua et al., in a comprehensive review, emphasized that surgical release remains the gold standard for managing persistent CTS, offering substantial symptomatic relief, improved nerve conduction, and enhanced patient quality of life [15].

Limitations of this study

This study has several limitations. It was conducted at a single center, which may limit the generalizability of the findings. The sample size was relatively small (n=50), potentially affecting the statistical power of the results. Additionally, the follow-up period was short (six months), which may not capture long-term outcomes. Lastly, the absence of a control group continuing conservative therapy restricts the ability to directly compare surgical outcomes with non-operative management.

## Conclusions

CTR is a safe and effective surgical option for patients with persistent CTS unresponsive to conservative treatment, offering significant pain relief, improved nerve conduction, and high patient satisfaction. In this study, 94% of the patients reported substantial symptom relief, with an 80% reduction in pain scores and notable improvements in nerve conduction velocity. CTR also enhanced occupational outcomes, with a marked reduction in work-related disability and a mean return-to-work time of just over 11 days. The procedure had a low complication rate, limited to mild, self-resolving issues.

## Appendices

 Boston Carpal Tunnel Questionnaire (BCTQ) 

The Boston Carpal Tunnel Questionnaire (BCTQ) is a validated, disease-specific tool used to assess the severity of symptoms and functional impairment in patients with carpal tunnel syndrome. It consists of two parts: the Symptom Severity Scale (SSS) and the Functional Status Scale (FSS). Each item is scored from 1 (no symptoms/difficulty) to 5 (severe symptoms/extreme difficulty), with higher scores indicating greater disability. A decrease in post-operative scores reflects clinical improvement following intervention.

**Table 5:** Symptom Severity Scale

Item	Question	Response Options (1–5)
SSS1	How severe is the hand or wrist pain that you have at night?	1 = No pain 2 = Mild pain 3 = Moderate pain 4 = Severe pain 5 = Very severe pain
SSS2	How often did hand or wrist pain wake you up during a typical night?	1 = Never 2 = Once 3 = Two or three times 4 = More than three times 5 = I wake up every hour
SSS3	Do you typically have pain in your hand or wrist during the daytime?	1 = No pain 2 = Mild pain 3 = Moderate pain 4 = Severe pain 5 = Very severe pain
SSS4	How often do you have hand or wrist pain during the daytime?	1 = Never 2 = Occasionally 3 = Frequently 4 = Very frequently 5 = Constantly
SSS5	How long, on average, does an episode of pain last during the day?	1 = No pain 2 = <10 min 3 = 10–60 min 4 = >1 hour 5 = Continuous
SSS6	Do you have numbness (loss of sensation) in your hand?	1 = No numbness 2 = Mild 3 = Moderate 4 = Severe 5 = Very severe
SSS7	Do you have weakness in your hand or wrist?	1 = No weakness 2 = Mild 3 = Moderate 4 = Severe 5 = Very severe
SSS8	Do you have tingling sensations in your hand?	1 = No tingling 2 = Mild 3 = Moderate 4 = Severe 5 = Very severe
SSS9	How severe is the numbness or tingling at night?	1 = None 2 = Mild 3 = Moderate 4 = Severe 5 = Very severe
SSS10	How often did hand numbness or tingling wake you up during a typical night?	1 = Never 2 = Once 3 = Two or three times 4 = More than three times 5 = I wake up every hour
SSS11	Do you have difficulty with grasping and using small objects such as keys or pens?	1 = No difficulty 2 = Mild 3 = Moderate 4 = Severe 5 = Cannot do at all

**Table 6:** Functional Status Scale (FSS)

Item	Activity	Response Options (1–5)
FSS1	Writing	1 = No difficulty 2 = Mild 3 = Moderate 4 = Severe 5 = Cannot do
FSS2	Buttoning clothes	1 = No difficulty 2 = Mild 3 = Moderate 4 = Severe 5 = Cannot do
FSS3	Holding a book while reading	1 = No difficulty 2 = Mild 3 = Moderate 4 = Severe 5 = Cannot do
FSS4	Gripping a telephone handle	1 = No difficulty 2 = Mild 3 = Moderate 4 = Severe 5 = Cannot do
FSS5	Opening jars	1 = No difficulty 2 = Mild 3 = Moderate 4 = Severe 5 = Cannot do
FSS6	Household chores	1 = No difficulty 2 = Mild 3 = Moderate 4 = Severe 5 = Cannot do
FSS7	Carrying grocery bags	1 = No difficulty 2 = Mild 3 = Moderate 4 = Severe 5 = Cannot do
FSS8	Bathing and dressing	1 = No difficulty 2 = Mild 3 = Moderate 4 = Severe 5 = Cannot do
